# Relative contributions of lesion location and lesion size to predictions of varied language deficits in post-stroke aphasia

**DOI:** 10.1016/j.nicl.2018.10.017

**Published:** 2018-10-19

**Authors:** Melissa Thye, Daniel Mirman

**Affiliations:** a Department of Psychology, University of Alabama at Birmingham, Birmingham, AL, USA; b Moss Rehabilitation Research Institute, Elkins Park, PA, USA

**Keywords:** Aphasia, Lesion-symptom prediction, Sparse canonical correlation analysis, Lesion size

## Abstract

Despite the widespread use of lesion-symptom mapping (LSM) techniques to study associations between location of brain damage and language deficits, the prediction of language deficits from lesion location remains a substantial challenge. The present study examined several factors which may impact lesion-symptom prediction by (1) testing the relative predictive advantage of general language deficit scores compared to composite scores that capture specific deficit types, (2) isolating the relative contribution of lesion location compared to lesion size, and (3) comparing standard voxel-based lesion-symptom mapping (VLSM) with a multivariate method (sparse canonical correlation analysis, SCCAN). Analyses were conducted on data from 128 participants who completed a detailed battery of psycholinguistic tests and underwent structural neuroimaging (MRI or CT) to determine lesion location. For both VLSM and SCCAN, overall aphasia severity (Western Aphasia Battery Aphasia Quotient) and object naming deficits were primarily predicted by lesion size, whereas deficits in Speech Production and Speech Recognition were better predicted by a combination of lesion size and location. The implementation of both VLSM and SCCAN raises important considerations regarding controlling for lesion size in lesion-symptom mapping analyses. These findings suggest that lesion-symptom prediction is more accurate for deficits within neurally-localized cognitive systems when both lesion size and location are considered compared to broad functional deficits, which can be predicted by overall lesion size alone.

## Highlights

• Lesion location improves prediction for speech production and speech recognition.
• Broad deficits, aphasia severity and naming, are primarily predicted by lesion size.
• Lesion location may be more informative for neurally-localized cognitive systems.
• Predictive inference is an alternative way to control for lesion size.

## 1 Introduction

Aphasia is an impairment of language that occurs in up to 46% of stroke survivors and is associated with substantial negative effects on health and quality of life, including reduced participation in activities across all domains of daily life and increased likelihood of death within 2 years of stroke (Boehme et al., 2016; Flowers et al., 2016; Hilari, 2011). Although many patients recover some degree of language function, recovery is highly variable (Lazar et al., 2008; Pedersen et al., 2004). Lesion characteristics such as size and location may differentially contribute to this variability in language recovery outcomes and can be studied using lesion-symptom mapping (LSM), a key method for examining the association between lesion site and performance on a language task or battery of tasks (Bates et al., 2003).

Recent studies have attempted to use this LSM approach for predicting outcomes in patients with post-stroke aphasia (Price et al., 2010), with mixed results. One study found that demographic information, lesion size, and atlas-based lesion location predictors accounted for almost 60% of the variance in a composite speech production score (Hope et al., 2013). Another group used a machine learning (support vector machine) approach to classify patients into aphasia subtypes using the percentage of damage within atlas-derived regions (Yourganov et al., 2015), but achieved above-chance performance for only 5–7 of 10 binary classifications. A more recent study used random forests with a multimodal combination of structural lesion data and functional and structural connectivity data (Pustina et al., 2017b) to account for nearly 80% of the variance on the Philadelphia Naming Task (PNT) and composite measures from the Western Aphasia Battery (WAB). These studies are promising, though they vary in the kind of lesion data used and the type of deficits predicted. The latter issue may be particularly important as one group highlighted the utility of using a composite score comprised of several measures assessing the same domain of language (speech production) compared to individual scores from single-item measures (Hope et al., 2013).

Several recent studies have combined principle component analysis (PCA) and lesion-symptom mapping to identify the core systems of language processing and relate deficits in these systems to lesion location in patients with post-stroke aphasia (for a review see Mirman and Thye, in press). This approach allows for the identification of the neural basis of dissociable functional language systems, which may provide a stronger basis for lesion-symptom prediction. However, no lesion-symptom prediction study to date has examined the relative predictive advantage of single-measure scores from comprehensive language tasks versus composite scores derived from a battery of tasks assessing domain-specific language systems. Thus, one goal of the current study was to compare prediction of PCA-derived language sub-system deficit scores and more general language deficit scores.

Lesion-symptom prediction studies also need to carefully consider the role of overall lesion size. Individuals with larger left hemisphere lesions tend to perform worse on all language tasks, and larger lesions are more likely to impact a greater number of brain regions. Thus, overall lesion size affects both sides of the lesion-symptom prediction equation. In order to isolate these effects of lesion size, the second goal of the current study was to investigate the relative contribution of lesion location compared to lesion size. If lesion size alone is the best predictor, then the best course for evaluating prognosis is to use lesion size as a coarse measure of severity. Conversely, if lesion location provides additional predictive information beyond lesion size, then lesion location may be helpful in designing individualized treatment plans.

The third goal of the current study was to compare standard voxel-based lesion-symptom mapping (VLSM) and multivariate LSM methods in the context of lesion-symptom prediction. Standard VLSM is a mass-univariate method that independently tests the association between damage in each voxel and a behavioral symptom score. There are several limitations inherent to this traditional VLSM approach, including possible mis-localization of lesion-symptom associations, difficulty capturing network effects of multiple brain regions, and bias toward regions that are more frequently damaged within the sample of participants (Inoue et al., 2014; Mah et al., 2014; Sperber and Karnath, 2017). Multivariate LSM methods have been developed to address these issues by simultaneously considering the association between behavioral deficits and the full lesion pattern. In this study, we used multivariate LSM based on sparse canonical correlation analysis (SCCAN) (Avants et al., 2014; Pustina et al., 2017a). In SCCAN, the correlations among the lesioned voxels and the symptom of interest are optimized by modifying the weights attributed to each in order to maximize the brain-behavior association. SCCAN directly addresses several limitations of the mass-univariate VLSM approach, and therefore, provides a multivariate alternative for making predictive inferences about lesion-behavior associations.

The aim of Study 1 was to apply the SCCAN method to data previously used in VLSM analyses as a verification of its applicability to this type of data and as a precursor to its use in lesion-symptom prediction. It was expected that the results of the SCCAN analysis would converge with previous VLSM findings using these same deficit measures. The primary aim of Study 2 was to compare the predictive utility of lesion size and lesion location for broad measures of language functioning (naming, aphasia severity) and for measures that reflect dissociable functional language sub-systems (semantics, speech production, speech recognition). Critical lesion locations were based on both mass-univariate VLSM and multivariate SCCAN LSM. It was expected that lesion size would be particularly predictive for broad measures of language deficits due to the multi-determined nature of these assessments whereas lesion location would be more predictive for symptoms that align with the core systems of spoken language.

## 2 Data

The data were drawn from a large-scale study of language processing following left hemisphere stroke. Analyses of other language deficits using earlier subsets of the participants have been reported in several previous articles (Mirman et al., 2015b, Mirman et al., 2015a; Mirman and Graziano, 2013; Schwartz et al., 2012, Schwartz et al., 2011, Schwartz et al., 2009; Thothathiri et al., 2012; Walker et al., 2011), which also provide more detailed descriptions of the participants and imaging methods. The study was carried out in accordance with protocols approved by the Institutional Review Boards at the Einstein Healthcare Network and University of Pennsylvania School of Medicine.

The participants were 128 individuals with aphasia secondary to left hemisphere stroke (not bilateral or solely subcortical). To be included in this study, participants had to be at least 1 month post onset of aphasia secondary to stroke,^1^ living at home, medically stable without major psychiatric or neurological co-morbidities, no previous history of stroke, and premorbidly right handed. Participants were also required to have English as the primary language, adequate vision and hearing (with or without correction) and computed tomography (CT) or magnetic resonance imaging (MRI) confirmed left hemisphere cortical lesion. Participants completed a detailed battery of psycholinguistic tests which have been described in previous studies (Mirman et al., 2010) and are further described in the supplementary materials. Only participants who had completed all 17 measures used in our prior PCA LSM studies (Mirman et al., 2015b, Mirman et al., 2015a) were included in this study. Participant demographic information is presented in Table 1.

**Table 1:** Participant demographics.

	N	Mean (SD)	Range
Age	128	58.20 (11.68)	26–79
Years of Education	128	14.26 (2.97)	6–21
Lesion Size (cc)	128	100.97 (82.76)	5.38–376.12
Time Since Stroke (months)	128	51.59 (65.71)	1–381
WAB Aphasia Quotient	128	73.66 (19.38)	25.20–99.30
Gender (M:F)	71:57		
Aphasia subtype			
Anomic Aphasia	55		
Broca's Aphasia	31		
Conduction Aphasia	18		
Wernicke's Aphasia	10		
Transcortical Motor Aphasia	3		
Transcortical Sensory Aphasia	2		
Global Aphasia	1		
Other	8		

Note. N, number of participants; SD, standard deviation of the mean; WAB, Western Aphasia Battery; M, male; F, female.

**Fig. 1:**
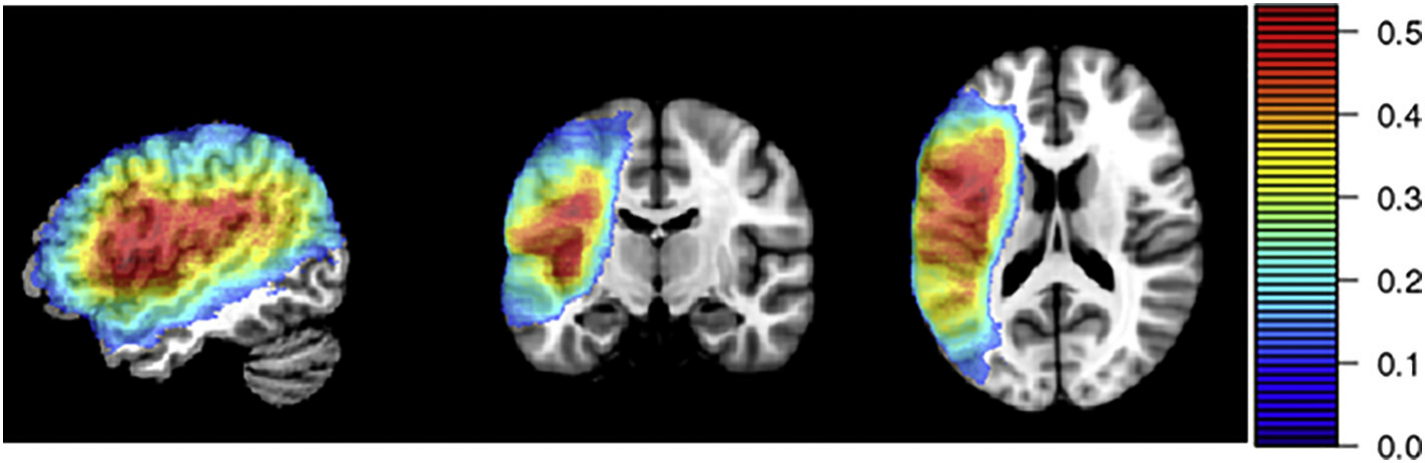
Lesion overlap for full sample of participants (*N* = 128). Hotter colors indicate voxels where a larger proportion of participants had lesions. Only regions where at least 10% of participants had lesions are included in the color map because only these voxels were included in the analyses.

Lesion location was assessed based on MRI (*n* = 75) or CT (*n* = 53) brain scans collected during the chronic stage (>6 months post onset) and following the same procedures as previous studies of this data set (or sub-sets of these data). For the MRI scans, lesions were manually segmented on each participant's T1-weighted structural image, then the structural scans and lesion maps were registered to the Montreal Neurological Institute (MNI) space Colin27 template by an automated process (Avants et al., 2006). For the CT scans, the lesion was drawn directly onto the Colin27 template by an expert neurologist after rotating it (pitch only) to match the approximate slice plane of the participant's scan. The lesion overlap map for the full sample of participants is shown in Fig. 1. Only structural lesion information was considered in the present study because this is the most widely available neural data for stroke survivors (because many are either unable or unwilling to undergo more sophisticated neuroimaging protocols) and because it most directly addresses the predictive utility of lesion size and lesion location. Multimodal neuroimaging (structural, connectivity, functional) would provide a more complete assessment of neural dysfunction after stroke and would almost certainly provide a more accurate deficit prediction, but the core principles related to lesion-symptom prediction explored here would remain the same.

**Fig. 2:**
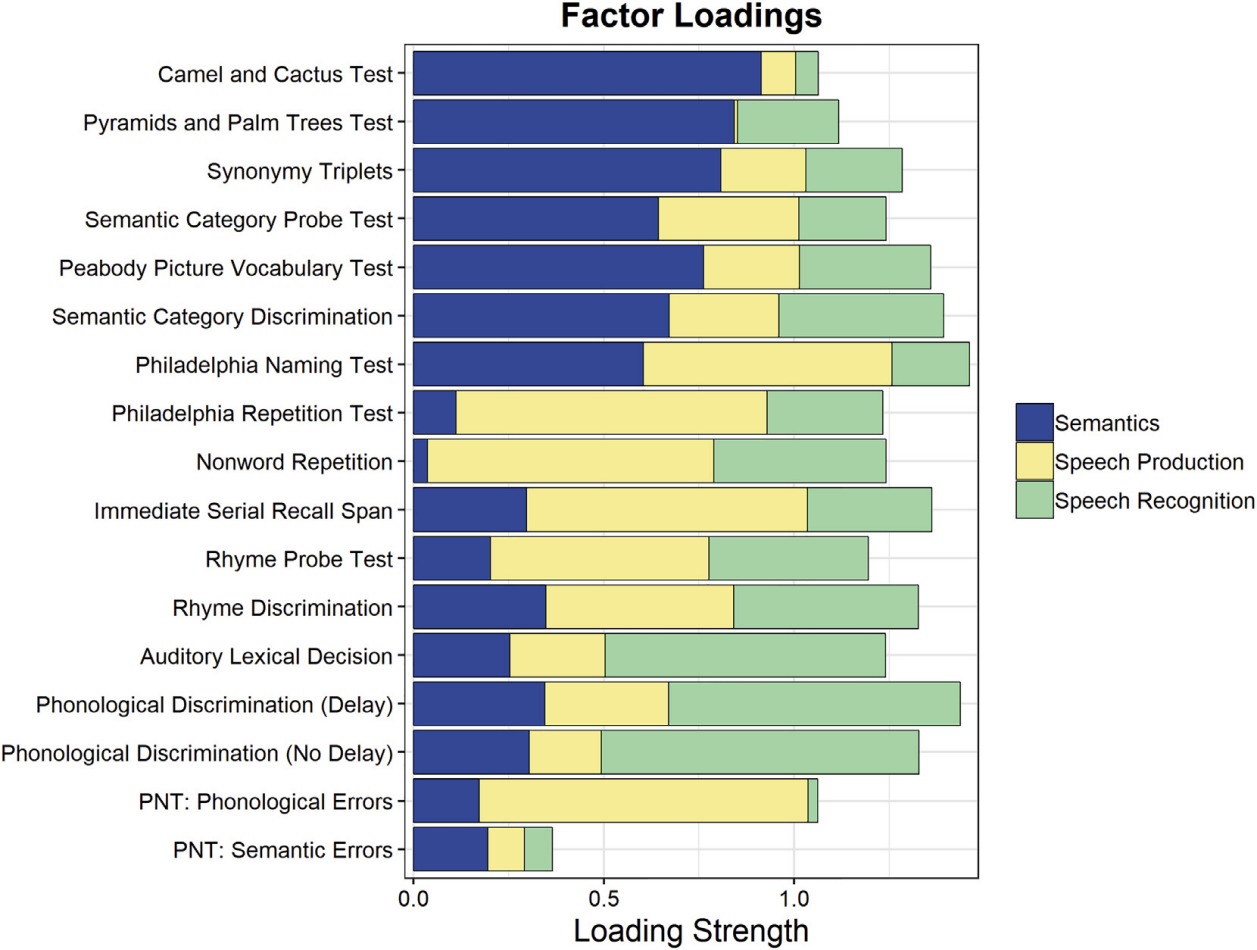
Factor loadings for the Semantics, Speech Production, and Speech Recognition factors from the principle component analysis run on 17 psycholinguistic measures.

As in our prior studies using PCA and LSM, participant scores on 17 psycholinguistic measures were entered into a principle component analysis with varimax rotation (Fig. 2; Supplemental Table). As reported in our previous studies (Mirman et al., 2015a, Mirman et al., 2015b), the four factor result corresponded to Semantic Recognition (e.g., Camel and Cactus Test, Synonymy Triplets), Speech Production (e.g., Philadelphia Repetition Test, Immediate Serial Recall Span), Speech Recognition (e.g., Phonological Discrimination, Auditory Lexical Decision), and Semantic Errors in picture naming and accounted for 27%, 24%, 19%, and 7% of the variance respectively. The Semantic Errors factor was not included in the subsequent analyses because it had an eigenvalue below 1.0 (0.915) and was characterized by a single high loading on semantic errors in picture naming, thus not representing a functional language sub-system in the same way as the other factors. The three factor solution explained 70% of the variance in the behavioral scores across participants. In addition to these three factor scores, overall picture naming ability (based on the Philadelphia Naming Test; PNT) and overall aphasia severity (Western Aphasia Battery Aphasia Quotient; WAB AQ) were used as general measures of language impairment.

## 3 Study 1

### 3.1 Methods

Lesion-symptom mapping analyses were conducted on the full sample of participants for each of the five deficit scores (PNT, WAB AQ, Semantics, Speech Production, and Speech Recognition) using the multivariate SCCAN method (Avants et al., 2014; Pustina et al., 2017a) implemented using the LESYMAP package for R (https://github.com/dorianps/LESYMAP). SCCAN relies on a sparseness parameter that determines the extent of voxels generated in the result. By default, this value is optimized using 4-fold cross-validation^2^ and the goodness of the overall LSM solution is assessed by cross-validated accuracy (CV correlation). Sparseness values range from 0 to 1 with larger values indicating less sparseness (i.e., a greater proportion of voxels retained) in the LSM solution. The sparseness value was separately optimized using this algorithm for each of the five deficit scores, for use in this Study and in Study 2. In addition, the lesion maps were normalized so an individual voxel with a value of 0 indicated no lesion within the voxel and 1/(sqrt of the total volume) indicated a lesion-damaged voxel while controlling for overall lesion size. This direct total lesion volume control method weights lesioned voxels from lesions with smaller volume more than voxels from lesions with larger volume (Mirman et al., 2015a; Zhang et al., 2014).

### 3.2 Results

**Fig. 3:**
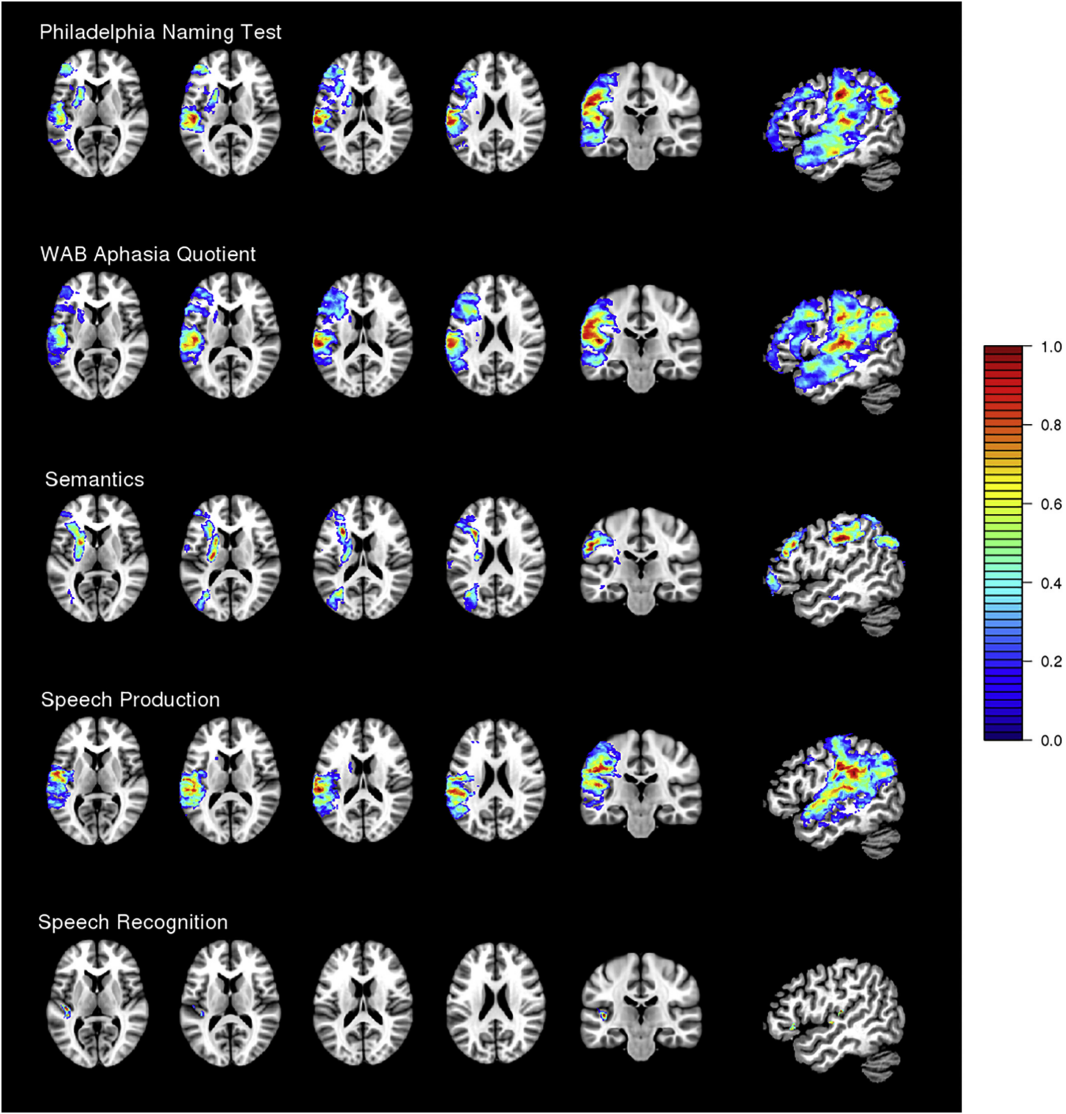
SCCAN results for the Philadelphia Naming Test (PNT), the Western Aphasia Battery Aphasia Quotient (WAB AQ), and the three PCA-derived functional language systems (Semantics, Speech Production, and Speech Recognition).

The values obtained from the sparseness optimization algorithm were 0.87 for PNT (CV correlation = 0.43), 0.88 for WAB AQ (CV correlation = 0.55), 0.68 for the Semantics factor (CV correlation = 0.32), 0.64 for the Speech Production factor (CV correlation = 0.45), and 0.02 for the Speech Recognition factor (CV correlation = 0.39). The SCCAN LSM results for each deficit score are shown in Fig. 3 (see also Table 2). A deficit in picture naming (lower accuracy on the PNT) was associated with damage in widespread portions of the middle cerebral artery (MCA) territory, including middle and inferior frontal regions as well as damage extending from anterior to posterior middle temporal regions and into the inferior parietal lobe (supramarginal gyrus, angular gyrus). Increased aphasia severity (lower WAB AQ) was similarly associated with damage to widespread middle and inferior frontal regions, and middle and superior temporal gyri extending posteriorly into parietal regions (supramarginal gyrus, angular gyrus).

**Table 2:** Percentage of each region obtained in the SCCAN results. All regions refer to left hemisphere.

Region	PNT	WAB AQ	Semantics	Speech Production	Speech Recognition
Total volume (number of voxels)	142,762	160,628	102,443	99,813	1500
Precentral gyrus	23.07	33.99	15.89	9.96	
Middle frontal gyrus	29.78	37.13	29.20	3.16	
Middle frontal gyrus (orbital part)	4.42	3.06	4.58		
Inferior frontal gyrus (pars opercularis)	35.27	61.61	9.60		
Inferior frontal gyrus (pars triangularis)	45.95	60.78	36.68		
Inferior frontal gyrus (pars orbitalis)	27.52	22.29	21.66	5.18	
Rolandic operculum	33.77	44.51		61.82	
Insula	17.07	22.45	24.19	31.35	
Superior occipital gyrus			3.62		
Middle occipital gyrus	9.72	5.71	36.91		
Postcentral gyrus	52.86	53.04	25.68	45.50	
Superior parietal gyrus			20.57		
Inferior parietal gyrus	32.81	39.78	53.40	28.17	
Supramarginal gyrus	80.75	89.68	16.76	91.02	
Angular gyrus	69.91	71.45	58.01	53.45	
Caudate	6.44	3.09	8.23	1.62	
Putamen	48.40	13.92	59.78	1.92	
Pallidum	1.73		21.38		
Heschl's gyrus	50.52	68.32		68.17	20.93
Superior temporal gyrus	77.12	86.83		87.55	3.35
Superior temporal pole	31.65	37.85		19.83	
Middle temporal gyrus	44.46	45.22		30.43	
Middle temporal pole	14.28	13.79			
Arcuate fasciculus	37.81	40.88	6.24	32.32	
Inferior fronto-occipital fasciculus	10.33	8.79	15.00	2.67	
Uncinate fasciculus	15.41	15.02	12.39	5.17	

Semantic deficits were associated with more focal damage to portions of the inferior and middle frontal gyrus, precentral gyrus, postcentral, angular, supramarginal, and superior parietal gyri, and portions of the insula, but not substantively including the temporal lobe. Damage to the underlying white matter, particularly the uncinate fasciculus, inferior fronto-occipital fasciculus, and arcuate fasciculus, was also associated with semantic deficits. Speech Production deficits were associated with damage to the supramarginal, angular, and postcentral gyri extending into the superior temporal lobe (superior temporal gyrus and Heschl's gyrus). Deficits in Speech Recognition were associated with highly localized damage to Heschl's gyrus. Because these analyses use composite behavioral deficit scores (factor scores), they reflect functional language systems and do not capture finer-grained divisions within these systems, which would be better captured by targeted deficit measures and are the subject of other studies (e.g., Schwartz et al., 2011, Schwartz et al., 2012; Thothathiri et al., 2012).

### 3.3 Discussion

The results of the SCCAN LSM analyses converged with previous findings using a mass-univariate VLSM approach, but also diverged in some intriguing ways. For the Speech Recognition deficit scores, SCCAN identified a very small region primarily in Heschl's gyrus. This is entirely consistent with prior LSM studies that have identified the left posterior superior temporal lobe as critical for speech recognition, but the identified region was far smaller than those reported in prior studies. For Speech Production deficit scores, the SCCAN results converged with prior LSM studies of speech production in identifying the “dorsal stream” (Hickok and Poeppel, 2007), primarily consisting of supramarginal, angular, and postcentral gyri, but the SCCAN results also included superior temporal regions. The SCCAN results for the Semantics factor included portions of inferior frontal and middle frontal gyri and insula, extending medially into the underlying white matter tracts. The convergence of the uncinate fasciculus and inferior fronto-occipital fasciculus has been previously characterized as a white matter bottleneck (Mirman et al., 2015b, Mirman et al., 2015a) where minimal damage incurred at the intersection of these critical tracts can result in significant semantic impairments. These brain-behavior associations reflect the primary functional systems involved in spoken language, which may have further sub-systems. For naming (PNT) and aphasia severity (WAB AQ), the breadth of the SCCAN results was even more striking, encompassing much of the MCA territory and showing little localization of lesion-symptom associations. In sum, SCCAN and mass-univariate VLSM tend to identify the same brain regions, but the extent of the identified regions can differ drastically.

The mass-univariate VLSM and SCCAN LSM approaches critically differ in how each method localizes the lesion-symptom associations. In mass-univariate VLSM, the lesion-symptom association is tested independently for each voxel, then a correction for multiple comparisons is applied. That correction can be computed in different ways (see Mirman et al., 2018), but all corrections share the property that voxels with stronger lesion-symptom associations will tend to survive the correction whereas voxels with weaker associations will not. How many voxels survive correction is strongly dependent on the conservativeness of the correction. For any given raw VLSM result, a conservative correction will leave a small “critical” region whereas a less conservative correction will leave a larger “critical” region.

In contrast, SCCAN LSM attempts to find the sparsest solution that optimizes the multivariate association between the lesion pattern and symptom severity. As a result, if damage in a small region is strongly associated with the symptom, then SCCAN will select a small optimal sparseness value and identify that small region. This is what we observed for the Speech Recognition deficit scores. However, if the lesion-symptom association is diffuse (and strong enough to be detectable), then SCCAN will select a large optimal sparseness value and identify a large region (the result for WAB AQ is the clearest example of this). In other words, the number of voxels included in the result is dependent on the sparseness/diffuseness of the lesion-symptom association, not on the conservativeness of the multiple comparisons correction. This difference has substantial implications for understanding the roles of overall lesion size and lesion location in lesion-symptom associations. Study 2 shed further light on this issue, and we will return to it after presenting those results.

## 4 Study 2

The goal of this study was to evaluate the predictive value of lesion size and lesion location for general language deficit scores (naming and aphasia severity) and more specific language sub-system deficit scores (PCA-derived scores for Semantics, Speech Production, and Speech Recognition). Two separate sets of analyses were conducted to compare the critical lesion locations derived from mass-univariate VLSM and SCCAN LSM.

### 4.1 Methods

**Fig. 4:**
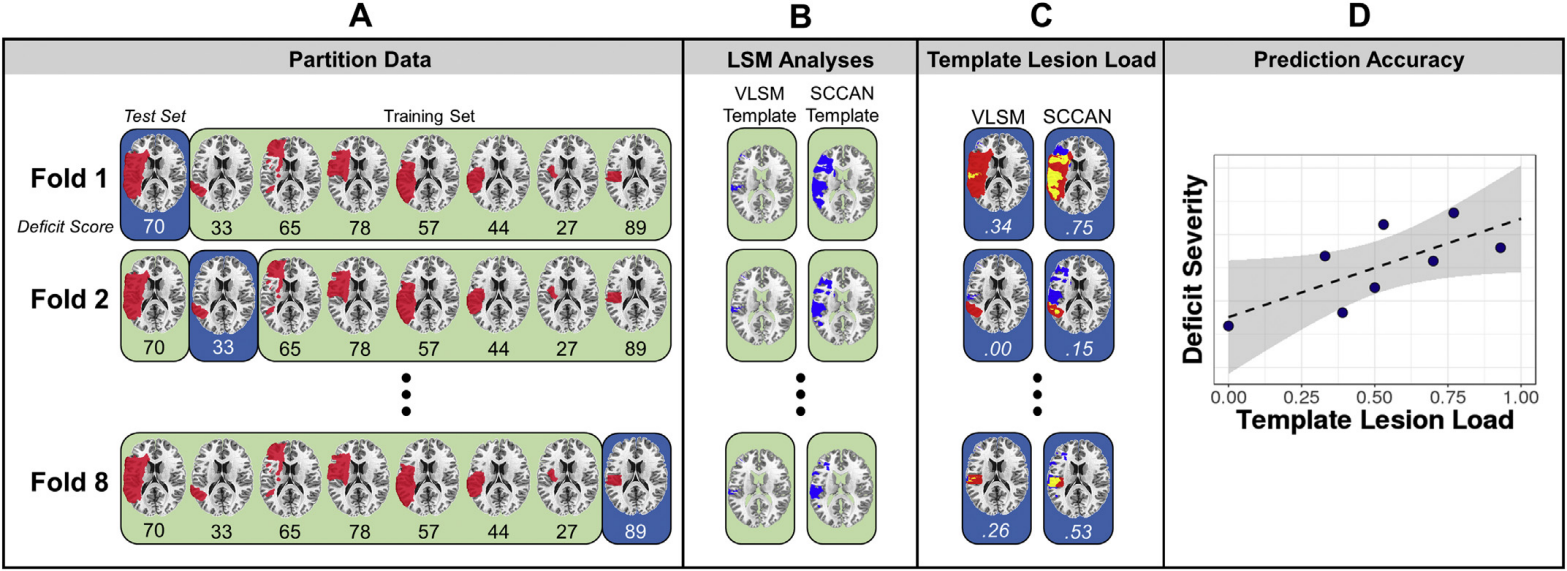
Schematic showing the analysis pipeline. Participant lesion and behavioral data were initially partitioned into 8 groups or “folds” (blue). For a given fold, the other folds served as the training set (green) for the subsequent LSM analyses (A). For each fold, VLSM and SCCAN were run on the training set of participants to generate a template of regions associated with the deficit (B). For each participant in the held-out test set, the template lesion load was calculated as the proportion of overlap between the LSM-generated templates and the participant's lesion (C). This process was repeated for all 8 folds to calculate template lesion load for each participant, which was then tested as a predictor of deficit severity (D). The schematic illustrates the full pipeline for one deficit score, and the pipeline was repeated for each of the five deficit scores.

Lesion-symptom prediction was implemented using 8-fold cross-validation. Participants were partitioned into 8 “folds” (*n* = 16 each) and, for each fold, LSM analyses were carried out for each of the deficit scores on the “training” data (*n* = 112). The mass-univariate VLSM’ results were corrected for multiple comparisons using permutation-based continuous FWER (Mirman et al., 2018) with v = 100. For consistency across folds, the full-sample optimal sparseness values from Study 1 were used in the SCCAN analyses. Both the VLSM and SCCAN analyses controlled for lesion size using the total direct lesion volume control described in Study 1 (Mirman et al., 2015a; Zhang et al., 2014). The LSM results were thresholded to generate a “template” of the critical brain regions associated with each deficit. For each participant in the held-out (“testing”) fold, template lesion load was generated by 1) calculating the overlap between the lesion and each template and 2) dividing this overlap by the total size of the template, thus accounting for the size of the template (which varied across behavioral scores and, to a smaller degree, across folds). That is, an individual's template lesion load is the proportion of the independently-generated LSM-based template that falls within that individual's lesion. Repeating this procedure for each of the 8 folds produced the two critical predictors – overall lesion size and template lesion load – for each participant. Stepwise regression was used to test the associations between each deficit score and lesion size, template lesion load, and template lesion load controlling for lesion size. A schematic of the analysis pipeline is shown in Fig. 4. All analyses were implemented in R using the LESYMAP package (https://github.com/dorianps/LESYMAP).

### 4.2 Results

**Fig. 5:**
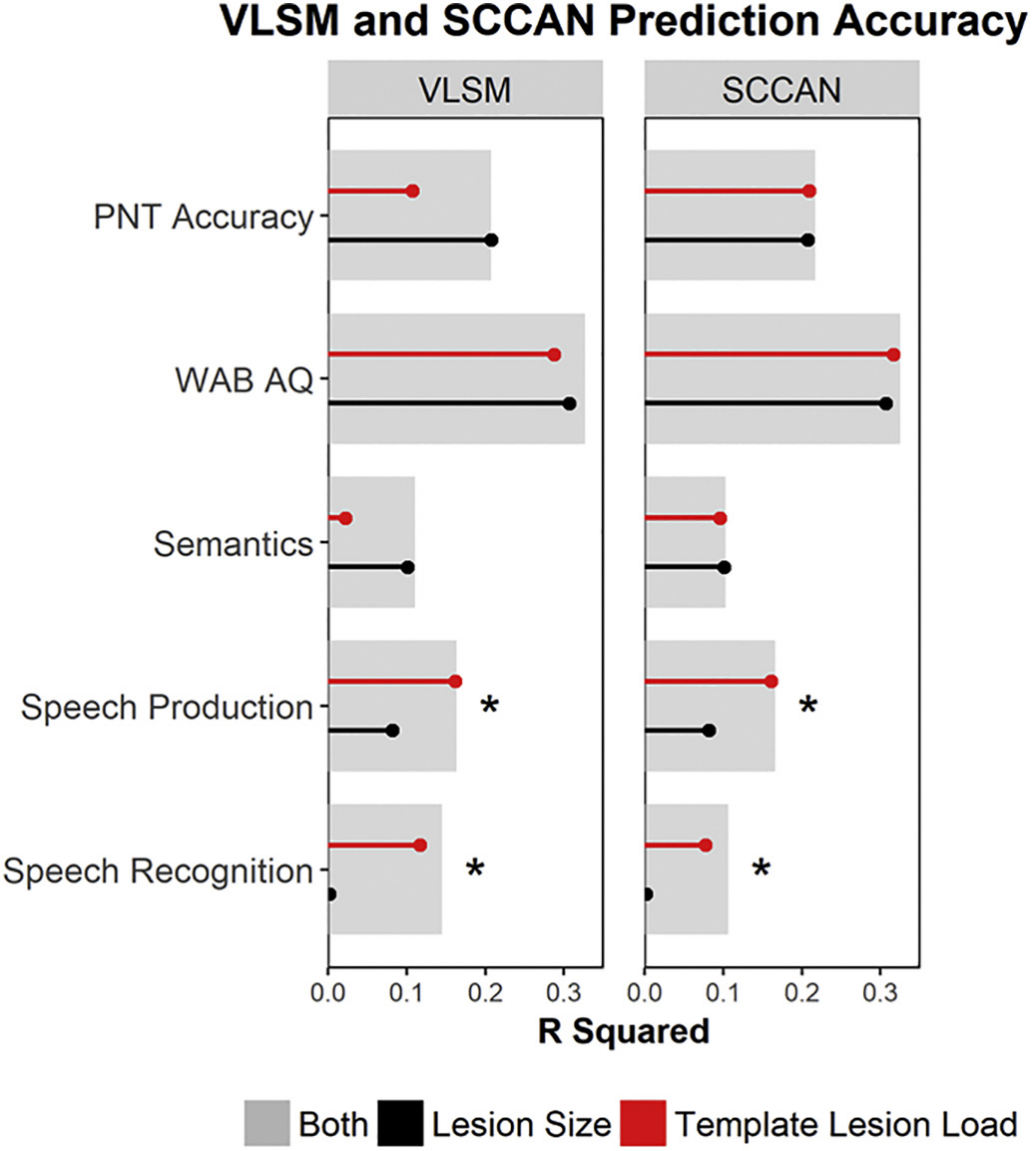
Prediction accuracy of VLSM and SCCAN method using lesion size (black), template lesion load (red), or both lesion size and template lesion load (grey) on scores on the Philadelphia Naming Test (PNT), the Western Aphasia Battery Aphasia Quotient (WAB AQ), and the three PCA-derived factors (Semantics, Speech Production, and Speech Recognition). * Indicates statistically significant (*p* < .05) increase in R^2^ for model with both predictors compared to model with only lesion volume.

The deficit scores were weakly to moderately associated with lesion size, with higher correlations observed for WAB AQ (*r* = −0.55, *p* < .01), PNT (*r* = −0.45, *p* < .01), and Semantics (*r* = −0.32, *p* < .01) and weaker correlations observed for Speech Production (*r* = −0.28*, p* < .01) and Speech Recognition (*r* = −0.04, *p* = .66). There was a high degree of correspondence between the VLSM and SCCAN results (Fig. 5). For both VLSM and SCCAN, lesion size alone was a significant predictor of PNT accuracy, WAB AQ, Semantics, and Speech Production (*p* < .01). The largest percentage of the variance explained by lesion size was seen for the broad deficit measures: WAB AQ (31%) and PNT (21%). This did not hold for the PCA-based deficit measures: across both types of LSM, lesion size accounted for 10%, 8%, and 0.1% of the variance in scores on Semantics, Speech Production, and Speech Recognition, respectively, suggesting that lesion size did not explain as much variability in performance for these functional language systems.

Template lesion load was a significant predictor of PNT accuracy, WAB AQ, Speech Production, and Speech Recognition for both lesion-symptom mapping analyses (*p* < .01). For the SCCAN analysis, template lesion load also significantly predicted Semantics (*p* < .01). After controlling for lesion size, the association between deficit scores and the template lesion load had stronger effects for the Speech Production (VLSM: *r* = −0.20, *p* < .05; SCCAN: *r* = −0.26, *p* < .01) and Speech Recognition (VLSM: *r* = −0.23, *p* < .05; SCCAN: *r* = −0.28, *p* < .01) systems and weaker effects for Semantics (VLSM: *r* = 0.13, *p* = .15; SCCAN: *r* = −0.16, *p* = .07), WAB AQ (VLSM: *r* = −0.04, *p* = .66; SCCAN: *r* = −0.16, *p* = .09), and PNT (VLSM: *r* = −0.02, *p* = .79; SCCAN: *r* = −0.02, *p* = .85). In other words, after accounting for overall lesion volume, adding template lesion load improved prediction accuracy only for Speech Production and Speech Recognition deficit scores.

**Fig. 6:**
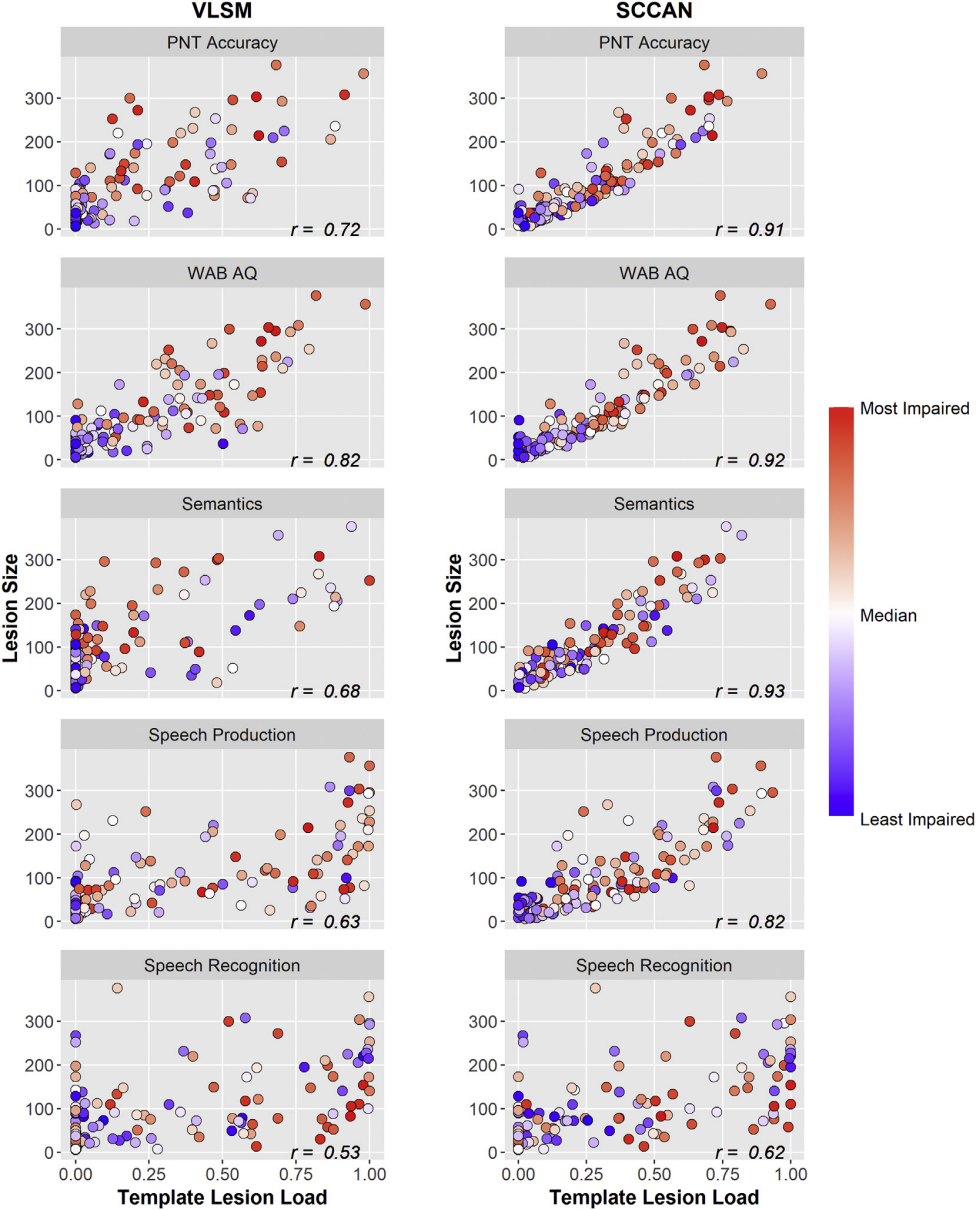
Relationship between lesion size and template lesion load for VLSM and SCCAN analyses for each deficit score. Participant scores are coded such that scores indicating a higher level of impairment within each domain are in red and scores indicating a lower level of impairment are in blue; PNT, Philadelphia Naming Test, WAB AQ, Western Aphasia Battery Aphasia Quotient; r, correlation coefficient.

To further explore this finding and to investigate what might be driving the influence of lesion size relative to template lesion load, severity scores for each deficit were plotted in relation to lesion size and template lesion load (Fig. 6). In general, participants with more severe deficits tended to have larger lesions and greater template lesion load. This was especially evident in the SCCAN results where there were moderate to strong correlations between lesion size and template lesion load across all deficit scores.

### 4.3 Discussion

For deficits in two of the functional language systems, Speech Production and Speech Recognition, lesion size alone was a poor predictor of outcomes and adding lesion location (overlap with a “critical” location template) to a lesion size only model significantly improved prediction accuracy. For general measures of spoken language performance, such as the Philadelphia Naming Test and the WAB Aphasia Quotient, overall lesion size and lesion location (overlap with a “critical” location template) were significant predictors of deficit severity, but lesion location did not significantly improve prediction accuracy above and beyond lesion size. This general pattern held regardless of whether the critical lesion locations were defined by mass-univariate VLSM or by multivariate SCCAN. The association between lesion size and PNT accuracy and WAB AQ is unsurprising given the multi-determined nature of these assessments; both measures draw on several components of spoken language, so there are multiple ways that performance can break down and possibly more opportunities for reorganization of function. Conversely, Speech Production and Speech Recognition seem to rely on more compact neural systems and may be less amenable to reorganization, so damage in particular critical brain regions will more consistently produce deficits in these domains. In other words, for these domains, knowing the size and location of the lesion results in greater prediction accuracy.

Semantic deficits had the lowest prediction accuracy, with only about 10% of the variance predicted from lesion information, and lesion location had no additional predictive utility beyond overall lesion size. Semantic cognition is supported by a bilateral distributed neural system with integrative hubs in the anterior temporal lobes and temporo-parietal cortex (especially angular gyrus), and critical involvement of frontal control systems and white matter tracts (e.g., Binder and Desai, 2011; Lambon Ralph et al., 2017; Mirman et al., 2015a, Mirman et al., 2015b). As a result, semantic deficits can arise due to damage to various sub-components of the distributed semantic system, and LSM may produce diffuse “templates” and a coarse measure such as template lesion load may not capture whether critical semantic processing components were damaged or not. In addition, semantic deficits can present in different ways, especially in left hemisphere stroke cases (e.g., Mirman and Britt, 2014), and may be more prone to re-organization and recovery, making them less predictable from structural lesion information.

Previous research has suggested that both lesion size and lesion site are important predictors of post-stroke aphasia recovery (Plowman et al., 2012). However, the utility of lesion location information appears to be dependent on the language process of interest. The size of a left middle cerebral artery (MCA) stroke lesion is expected to impact language performance, and there is little doubt that large lesions are likely to result in widespread language deficits. The critical theoretical claim, therefore, is whether, beyond lesion size, lesion location can be used to predict language performance and, beyond this, whether location is more informative for predicting specific language deficits. For the broad language measures, lesion location was not meaningfully different from lesion size given that the generated templates were large and the two measures were highly correlated (especially in the SCCAN version). Lesion location, therefore, was only as informative as lesion volume in the predictive models because it served as a proxy for lesion size. In the case of the broad deficit scores, the fact that lesion location does not improve prediction accuracy beyond that obtained with lesion size alone is essentially failing to reject the null hypothesis that the language deficit is a result of lesion size. For the specific scores (e.g., Speech Production and Speech Recognition), predictive models that included both lesion size and lesion location preformed significantly better than a model containing only the lesion size variable indicating that lesion location information does improve prediction accuracy. Prediction accuracy for speech production and recognition may be improved with the addition of lesion location due to the neuroanatomical specificity associated with each process. According to the dual-stream model of speech processing, language is functionally organized into two complementary processing streams: a bilateral ventral speech recognition stream extending from posterior portions of the superior and middle temporal gyri to anterior portions of the middle temporal gyrus, and a left hemisphere dorsal speech production stream which projects from the posterior superior temporal gyrus into inferior frontal and premotor areas (Hickok and Poeppel, 2007). This organization has been confirmed by studies using PCA and lesion-symptom mapping (Fridriksson et al., 2016; Mirman et al., 2015b, 2015a). The consistency of reported factors and neural basis of each functional language system across studies suggests that these functional subdivisions are robust. Thus, lesion location may be informative for these language sub-systems, which are supported by consistent and distinct neuroanatomy.

In contrast, aphasia severity (WAB AQ) and naming deficits (PNT) were predictable from overall lesion size, but lesion location provided no additional predictive utility. This is not surprising considering the multi-determined nature of these assessments. However, in each of these analyses, lesion locations associated with each deficit were defined while controlling for overall lesion size, and yet, overall lesion size remained the primary predictor of deficit severity. A model containing both lesion size and lesion location only had additional predictive power for two of the deficit scores: Speech Recognition and Speech Production. Template lesion load was strongly correlated with overall lesion size, particularly for SCCAN, where the large sparseness values for PNT, WAB AQ, and Semantics meant that the lesion-deficit “template” was not very different from the MCA territory. In other words, the sparseness optimization algorithm in SCCAN settled on a large sparseness value precisely because, for these measures, lesion size was a strong predictor of deficit severity and lesion location was not. For mass-univariate VLSM, the details of the data are the same (lesion size is the primary predictor of deficit scores or PNT, WAB AQ, and Semantics; lesion location is a stronger predictor for Speech Production and Speech Recognition), but the analysis reveals this in a somewhat different way. The template size is based on correction for multiple comparisons and only the voxels with the strongest lesion-symptom association survive that correction. However, damage to these voxels is revealed to have no unique predictive value over lesion size for PNT, WAB AQ, and Semantics deficits. In other words, when overall lesion size is the primary predictor of deficit severity, this is inherent in the SCCAN LSM result (large optimal sparseness value, large resulting region), but is obscured by multiple comparisons correction in mass-univariate VLSM and only emerges upon cross-validation testing.

## 5 General discussion

This study examined the prediction of different language deficits following left hemisphere stroke based on lesion size and lesion location. Critical lesion locations for each deficit were determined by creating templates using VLSM or SCCAN LSM. The critical findings were (1) Speech Production and Speech Recognition deficits were better predicted by a model containing both lesion size and lesion location, whereas general language deficits (aphasia severity and naming deficits) were predicted by lesion size and lesion location did not improve these predictions; and (2) SCCAN LSM inherently captured this by choosing a large optimal sparseness value for WAB AQ and PNT, which produced very large lesion location “templates”, whereas for VLSM, the size of the lesion location templates was determined by the multiple comparisons correction and the (lack of) predictive utility of lesion location only emerged in a subsequent cross-validation predictive analysis.

The SCCAN sparseness optimization algorithm iteratively alters the weights applied to the provided multidimensional neuroimaging dataset and the behavioral scores until a sparseness value that optimizes the association between these sources of data has been identified (Avants et al., 2014; Pustina et al., 2017a). This value determines the sparseness of the resulting statistical map (e.g., associated voxels) with smaller sparseness values resulting in a sparser solution. The sparseness values obtained after optimization were relatively high for the PNT, WAB AQ, and Semantics scores suggesting that a greater number of voxels were required in order to optimize the relationship between the lesion site and these behavioral scores. In other words, the relationship between the behavioral scores and the lesioned voxels was best described by a solution that encompassed a larger area extending across the left hemisphere regions affected by the lesion. This can be seen in Fig. 3, where the results obtained for PNT, WAB AQ, and Semantics are relatively distributed across language regions within the left hemisphere. The PNT and WAB AQ results, in particular, may be driven by the nature of these assessments, which draw on several language abilities. Widespread recruitment of frontal and parietal regions seen for the Semantics factor may reflect the distributed nature of semantic processing, which relies on a hub-and-spoke neural architecture, and the particular relevance of cognitive control deficits for semantic impairments in post-stroke aphasia (Lambon Ralph et al., 2017).

The sparseness values obtained for the Speech Production and, in particular, the Speech Recognition factors were much smaller compared to the other probed domains. The critical neuroanatomy of speech production and speech recognition appears to be relatively localized; thus, a solution with fewer voxels can optimally capture the association between the behavioral scores and the lesion location. The SCCAN results for the Speech Production and Speech Recognition factors largely encompassed regions within the dorsal and ventral routes of speech processing (Hickok and Poeppel, 2007). Interestingly, the SCCAN result for Speech Recognition was highly localized to Heschl's gyrus which may reflect an early auditory processing component of speech recognition. Because SCCAN is designed to find the sparsest optimal solution, this result may be a particularly important sub-component of the ventral speech comprehension system that has been identified in previous studies that used PCA in combination with mass-univariate and SVR-LSM methods (Fridriksson et al., 2016; Mirman et al., 2015a, Mirman et al., 2015b).

Previous lesion-symptom prediction studies have achieved relatively good prediction accuracy through the inclusion of lesion size, lesion location, patient demographic information, atlas-based regional information, and even multimodal neuroimaging information into the model (Hope et al., 2013; Pustina et al., 2017b; Yourganov et al., 2015). The current study isolated the impact of lesion location relative to lesion size by excluding other variables or sources of information. This was done to rigorously examine the relative contributions of lesion size compared to lesion location across a range of behavioral scores and outcomes. In addition, assessing model performance using these more selective sources of data represents a scenario that is likely to occur in a clinical setting where multimodal, atlas-derived metrics may not be available. As a result, the modest amount of variance in deficit severity explained by the models represents a lower bound on what can be accomplished with simple models based on lesion size and location alone. Multimodal neuroimaging information, when available, is likely to improve model prediction accuracy and can provide more detailed information about the neural basis of language and its sub-systems. Other factors, including demographic (e.g., age), social (e.g., social support), and personality (e.g., optimism) differences, are also likely to contribute to individual differences in recovery.

The present study raises important concerns about what it means to “control for lesion size” in lesion-symptom mapping analyses. The VLSM analyses reported here controlled for lesion size at the voxel level and yet damage to regions identified for WAB AQ, PNT, and Semantic deficit was no more related to those deficits than overall lesion size was. One interpretation of this result is that the lesion size control was not sufficiently effective and a more conservative, behavior-side control is necessary (i.e., using residual behavioral deficit scores after controlling for lesion size). Indeed, this would more conservatively control for lesion size by attributing as much of the behavioral deficit variability to lesion size as possible. In an additional analysis, we found that using such a behavior-side control for lesion size did effectively eliminate effects for PNT and WAB AQ, which was expected given the role of lesion size in predicting deficits in these domains. However, the behavior-side control also eliminated large portions of the effects for the other deficits suggesting that (1) the behavior-side control may be overly conservative, producing false negatives, and (2) lesion location is critical for predicting deficit scores for Speech Production and Speech Recognition even when using an overly stringent lesion-size correction method. A detailed evaluation of different lesion size control methods would require additional large-scale simulation studies of both univariate and multivariate LSM analyses in which the ground truth is known. Recent studies of this sort (DeMarco and Turkeltaub, 2018; Sperber and Karnath, 2017; Xu et al., 2018) clearly demonstrate that lesion volume control is critical for accurate mapping of lesion-symptom associations, though the relative advantages of different control methods in the context of different LSM methods may not be easy to identify. More generally, the value of simulation studies depends on how well the simulations capture actual lesion and deficit distributions, and should converge with studies using real data (Xu et al., 2018). Predictive inference, as used in the cross-validation analyses in Study 2, appears to provide an alternative approach that effectively controls for lesion size by testing whether the LSM result has a significant effect on prediction accuracy after using lesion size as a control variable. SCCAN LSM implements a version of this kind of strategy in its sparseness optimization process, which uses cross-validation prediction accuracy and a bias toward sparser solutions to identify a sparseness value which optimally captures the relationship between the lesion and the deficit score. As a result, in cases where lesion size is a significant predictor and lesion location is not, SCCAN produces a large sparseness value and broadly extended LSM result, implicitly identifying overall lesion size as the critical predictor of deficit severity.

The present study found that deficits in two functional language systems, speech production and speech recognition, were better predicted by lesion location in addition to lesion size. This suggests that lesion-symptom prediction is more accurate for deficits within neurally-localized cognitive systems than for broad functional deficits, which may be better predicted by overall lesion size. As researchers begin to focus more on lesion-symptom prediction, the present results suggest that it is important to carefully select the deficits for prediction. Some deficit measures may be so broad that they are effectively predicted by overall lesion size and sophisticated ways to identify “critical” brain regions will have little additional predictive utility. In contrast, focusing on functional cognitive systems that support language and rely on more consistent and dissociable neural systems may produce more accurate deficit predictions. Such predictions could be useful to clinicians and therapists for targeting suspected or predicted deficits, individualizing treatment planning, and ultimately improving long-term outcomes.

## Appendix A Supplementary data

Additional information about principal component analysis and the psycholinguistic battery. Image 1

## Footnotes

1 The vast majority of participants were tested in the chronic stage (98% were at least 3 months post onset). Exclusion of participants who were not in the chronic stage did not substantively alter any of the reported results, so the more inclusive analyses are reported here.

2 In pilot testing, we found that the optimization algorithm was robust to its parameters, such as number of folds, converging to essentially the same sparseness value across a range of parameter values.
