# Supplementary material for: Relative contributions of lesion location and lesion size to predictions of varied language deficits in post-stroke aphasia
Source: Neuroimage Clin. 2018 Oct 19;20:1129–38. doi: 10.1016/j.nicl.2018.10.017 (PMC6205357; doi:10.1016/j.nicl.2018.10.017)
Supplement: Supplementary file 1 — Additional information about principal component analysis and the psycholinguistic battery. [file mmc1.docx]

Supplementary Materials

Supplemental Table

*Factor Loadings from Principle Component Analysis*

|  | Semantics | Speech Production | Speech Recognition |
| --- | --- | --- | --- |
| Camel and Cactus Test | 0.914 | 0.090 | 0.059 |
| Pyramids and Palm Trees Test | 0.842 | 0.008 | 0.267 |
| Synonymy Triplets | 0.808 | 0.223 | 0.254 |
| Semantic Category Probe Test | 0.623 | 0.370 | 0.228 |
| Peabody Picture Vocabulary Test | 0.762 | 0.252 | 0.345 |
| Semantic Category Discrimination | 0.671 | 0.289 | 0.433 |
| Philadelphia Naming Test | 0.603 | 0.655 | 0.203 |
| Philadelphia Repetition Test | 0.111 | 0.818 | 0.304 |
| Nonword Repetition | 0.036 | 0.753 | 0.453 |
| Immediate Serial Recall Span | 0.296 | 0.739 | 0.326 |
| Rhyme Probe Test | 0.202 | 0.575 | 0.419 |
| Rhyme Discrimination | 0.348 | 0.494 | 0.485 |
| Auditory Lexical Decision | 0.254 | 0.250 | 0.736 |
| Phonological Discrimination (Delay) | 0.345 | 0.326 | 0.766 |
| Phonological Discrimination (No Delay) | 0.304 | 0.189 | 0.836 |
| PNT: Phonological Errors | -0.173 | -0.864 | 0.025 |
| PNT: Semantic Errors | -0.195 | 0.096 | -0.073 |

**Psycholinguistic Battery**

Additional description of the psycholinguistic battery has been provided in previous reports using subsets of this data (Mirman et al., 2015a, 2010).

| **Test** | **Description** | **Mean (SD)** |
| --- | --- | --- |
| *Camel and cactus test* | A non-verbal test of semantic processing in which a pictured item must be matched to the closest associate among a set of four pictured choices. Performance is measured by percent correct of 64 trials. | 75.66 (14.71) |
| *Pyramids and palm trees test* | A non-verbal test of semantic processing in which a pictured item must be matched to the closest associate among a set of two pictured choices. Performance is measured by percent correct of 52 trials. | 87.61 (10.66) |
| *Synonymy triplets* | A verbal test of semantic processing in which participants must decide which two of three words are most similar in meaning. Half the trials involve nouns, the other half verbs. Performance is measured by percent correct of 30 trials. | 80.23 (16.54) |
| *Semantic category probe test* | Test of semantic short-term memory in which participants listen to a list of three or more words and must determine whether the final word is from the same category as any of the preceding words by saying or pointing to ‘Yes’ or ‘No’. The list of words gradually increases and performance is measured as the maximum list length with 75% or higher accuracy. | 2.23 (1.30) |
| *Peabody picture vocabulary test* | An untimed, norm-referenced spoken word-to-picture matching vocabulary test arranged in order of increasing difficulty and representing various parts of speech. Performance is measured by a standard score. | 79.09 (16.86) |
| *Semantic category discrimination* | A verbal test of semantic processing in which participants must indicate whether two spoken words are members of the same semantic category by saying or pointing to ‘Yes’ or ‘No’. Performance is measured by percent correct of 40 trials. | 83.72 (12.33) |
| *Philadelphia naming test* | A 175-item single-word picture naming test using black and white line drawings of minimal complexity and confusability. The target words cover a relatively wide range of word length, word frequency and semantic category. The pictures are all familiar objects with high name agreement. Three performance measures were included: overall percent correct, percent of semantic errors, and percent of phonological errors. | Overall: 64.92 (28.87)  Semantic: 4.50 (3.18)  Phonological: 13.18 (13.40) |
| *Philadelphia repetition test* | A word repetition test using the same set of 175 targets as the Philadelphia naming test. Performance is measured by percent correct. | 86.00 (15.71) |
| *Nonword repetition* | Pre-recorded nonword targets derived from Philadelphia naming test target words were presented to participants for repetition. Performance is measured by percent correct of 60 trials. | 46.72 (26.13) |
| *Immediate serial recall span* | Test of short-term memory in which participants were required to repeat ten lists of one-syllable words, starting with two-word lists and increasing up to five-word lists, if possible. Performance is measured by span length of the form *X.Y*, where *X* is longest list with at least 50% correct recall and *Y* is the proportion correct on the next list out of 50%. | 2.78 (1.14) |
| *Rhyme probe test* | Test of phonological short-term memory in which participants listen to a list of three or more words and must determine whether the final word rhymes with any of the preceding words by saying or pointing to ‘Yes’ or ‘No’. The list of words gradually increases and performance is measured as the maximum list length with 75% or higher accuracy. | 2.90 (1.81) |
| *Rhyme discrimination* | Test of speech perception in which participants must indicate whether two spoken words rhyme by saying or pointing to ‘Yes’ or ‘No’. Performance is measured by percent correct of 30 trials. | 89.75 (11.72) |
| *Auditory lexical decision* | Test of spoken word recognition in which participants must indicate whether each item is a real English word or not (80 items of each type). Performance is measured as overall *d′* (a measure of discrimination based on signal detection theory). | 2.49 (0.73) |
| *Phonological discrimination* | Test of speech perception in which participants must indicate whether two spoken words or nonwords are the same or different. Non-identical pairs differ by a single onset or final phoneme. In the delay version, there is a 5s interval between the two items in a pair. Performance is measured by percent correct. | No delay: 88.50 (10.45)  Delay: 82.34 (12.20) |
